# Wearable CNTs-based humidity sensors with high sensitivity and flexibility for real-time multiple respiratory monitoring

**DOI:** 10.1186/s40580-022-00326-6

**Published:** 2022-08-01

**Authors:** Han-Sem Kim, Ji-Hye Kang, Ji-Young Hwang, Ueon Sang Shin

**Affiliations:** 1 Institute of Tissue Regeneration Engineering (ITREN), Dankook University, Cheonan, 31116 South Korea; 2 Department of Nanobiomedical Science & BK21 FOUR NBM Global Research Center for Regenerative Medicine, Dankook University, Cheonan, 31116 South Korea; 3 Convergence Research Division, Korea Carbon Industry Promotion Agency (KCARBON), Jeonju, 54853 South Korea

**Keywords:** Wearable humidity sensor, Flexible sensor, Core–shell structure, Resistive type sensor, Multiple respiratory monitoring

## Abstract

Sensors, such as optical, chemical, and electrical sensors, play an important role in our lives. While these sensors already have widespread applications, such as humidity sensors, most are generally incompatible with flexible/inactive substrates and rely on conventional hard materials and complex manufacturing processes. To overcome this, we develop a CNT-based, low-resistance, and flexible humidity sensor. The core–shell structured CNT@CPM is prepared with Chit and PAMAM to achieve reliability, accuracy, consistency, and durability, resulting in a highly sensitive humidity sensor. The average response/recovery time of optimized sensor is only less than 20 s, with high sensitivity, consistent responsiveness, good linearity according to humidity rates, and low hysteresis (− 0.29 to 0.30 %RH). Moreover, it is highly reliable for long-term (at least 1 month), repeated bending (over 15,000 times), and provides accurate humidity measurement results. We apply the sensor to smart-wear, such as masks, that could conduct multi-respiratory monitoring in real-time through automatic ventilation systems. Several multi-respiratory monitoring results demonstrate its high responsiveness (less than 1.2 s) and consistent performance, indicating highly desirable for healthcare monitoring. Finally, these automatic ventilation systems paired with flexible sensors and applied to smart-wear can not only provide comfort but also enable stable and accurate healthcare in all environments.

**Supplementary Information:**

The online version contains supplementary material available at 10.1186/s40580-022-00326-6.

## Introduction

Wearing of face masks by the general population has become a common and ubiquitous daily lifestyle worldwide. The daily wearing of personal protective equipment such as masks creates moisture inside owing to sweat and differences in internal and external temperature. Electrostatics-based masks are especially vulnerable to humidity, reducing the performance of the mask, and causing not only discomfort and nervous temperament to the wearer but also harm to the skin [1, 2].

Recently, many researchers have made great efforts to eliminate this inconvenience, including sensors and ventilation systems with additional filters. However, most of the early wearable sensors added to various accessories have been focused on function and performance rather than user convenience [3, 4]. In addition, owing to the lack of batteries, wireless communication technology, and measurement efficiency, these were operated internally via various communication devices, and sensors were also based on existing medical sensors, which caused great inconvenience to users. While the target performance of these state-of-the-art sensors has already been reached, most sensors so far have generally been incompatible with flexible/variable substrates and have relied on conventional inorganic hard materials and complex fabrication processes [5]. Multi-functional sensors with flexibility and scalability are rapidly being developed using various properties of organic materials, and the design of wearable sensors has become a major research topic. Wearable sensors can simplify daily life procedures and provide useful tools for on-site monitoring of internal and external parameters. These next-generation sensors must have low cost, low weight, and low energy to ensure compatibility with wearable technologies [6, 7].

To overcome this bottleneck, many researchers have been striving to develop more flexible and smaller bands, patches, clothing, and contact lenses that are easier for users to wear [8, 9]. Recently, the development of flexible electronic sensors has attracted interest for potential applications in industry and agriculture, as well as in personal healthcare, health assessment, and sports monitoring [10–14]. Meanwhile, flexible humidity sensors are rarely reported compared to other sensors because of a lack of recognition of their importance, despite humidity being one of the most intensively measured variables in our lives, as it is associated with a comfortable living environment, medical facilities, and body health information, among others [5]. However, flexible humidity sensors are promising candidates for potential applications in skin-like electronics, robotic human–machine interfaces, wearable electronic systems, soft robotics, and biomedical applications [15–18]. As a result, many studies on flexible humidity sensors are required. Humidity sensors work on two main operating principles: capacitive and resistive sensing methods [19]. Capacitive sensors are the most commonly used sensors and have the advantage of being quick and simple to react to; however, their accuracy is low owing to their large error range. Resistive sensors can be accurately measured; however, their measurement efficiency and response speed decrease depending on the range of resistance [20]. To compensate for the drawbacks of capacitive and resistive sensors, many studies have been conducted using carbon-based materials such as graphene, carbon nanotubes (CNTs), carbon black, and carbon fibers. However, despite many of these studies, flexibility and efficiency are limited by the rigidity and high resistance of the flexible humidity sensor [21, 22].

Among various materials to overcome the above limitations, the core–shell structural material has the most efficient structure competent of forming organic–inorganic nanocomposites that have attracted attention in various fields, including sensors [23]. Core–shell structured materials can be expected to have many advantages such as enhanced electrical properties, versatility, material stability, dispersibility, and minimized consumption of valuable materials based on a large surface part due to an efficient structure [24]. Therefore, it is the most attractive structure competent of overcoming the limitations of existing humidity sensors.

Here, we aim to develop a flexible humidity sensor that can measure precise humidity intuitively and stably by enabling humidity measurement with very fast and consistent resistance changes depending on the atmospheric humidity. To facilitate this, we employed a complex of chitosan (Chit) and PAMAM dendrimer G3 (PM) as an organic phase that acted as an adhesive to coat individual carbon nanotubes (CNTs) uniformly. The materials formed were characterized by a core–shell structure encased in numerous amines of Chit and PM, and these amines allowed for flexible properties and quick reactions with moisture through self-assembly. When applied to smart wear, such as face masks, we confirmed that real-time multi-respiratory monitoring and ventilation systems showed very accurate and consistent outcomes.

## Experimental section

### Materials

Pristine multi-walled carbon nanotubes (CNTs, > 95%, 20–30 nm of diameter, 10–30 μm of length) were purchased from Carbon Nanotech Co. (Pohang, Korea). CNTs were refluxed in a 5 N HCl solution for 1 d prior to use and washed in deionized water. All chemicals, chitosan (Chit, low molecular weight, 75–85% degree of deacetylation, 50–190 kDa), PAMAM dendrimer, ethylene core, generation 3.0 solution (PM), and including organic solvents, which were purchased from Sigma-Aldrich (St. Louis, MA, USA), were of analytical grade and used without further purification.

### Fabrication of PM-embedded Chit/CNT nanocomposites (CNT@CPM)

In order to fabricate CNT@CPM nanocomposites, each CNT was wrapped with PM-embedded Chit molecules in a core–shell structure that was prepared as follows: First, 45 mg of Chit was dissolved in 10 ml of acetic acid (1 v/v%) to make a Chit solution. 15 mg of CNT and each mol% of PM (Table 1) were then homogenized in the Chit solution using a homogenizer (MN600P-200, Micronox Corp., Seongnam, South Korea). This acidic CNT@CPM solution was slowly neutralized by 1 N ammonia solution, followed by dialysis membrane tube at a molecular weight cut-off of 12,000–14,000 Da (Spectrum Laboratories, Savannah, GA, USA) against deionized water for 3 d to remove any small molecules including organic and inorganic side products. The aqueous CNT@CPM solution showed a high degree of dispersion and stably with high hydrophilicity. (Additional file 1: Fig. S1).

**Table 1:** Code names of nanocomposites with different ratios of CNT, Chit, and PM

Code name	CNT (mg)	Chit (mg)	PM (mg / wt%)	Free amine ratio (times)
CNT@CPM-1	15	45	0 / 0	1
CNT@CPM-2			1.8 / 3	40.5
CNT@CPM-3			3.0 / 5	66.8
CNT@CPM-4			6.0 / 10	132.7

### Immobilization of CNT@CPM nanocomposites on the flexible polyimide surface of a gold electrode

CNT@CPM nanocomposites were immobilized on the gold electrode with a flexible polyimide surface. The flexible substrate was first washed ultrasonically in deionized water for 30 s. CNT@CPM nanocomposite solution (20 µL) was dropped on a gold electrode and dried at 60 °C. The resulting CNT@CPM nanocomposite-coated electrodes were thoroughly rinsed with water and absolute ethanol to remove any small molecules, including inorganic side products.

### Characterizations

The Chit, CNT, PM, and CNT@CPM nanocomposites were characterized through qualitative analysis using Fourier transform infrared spectroscopy (FT-IR Perkin-Elmer, Spectrum BXII FT-IR spectrometer, USA), which was repeated 16 times under potassium bromide pellet conditions over a spectral range of 400–4000 cm^−1^ at 4 cm^−1^ resolution. Raman spectroscopy (RAMAN DXR2xi, ThermoFisher, USA) was repeated 16 times under probing conditions over a spectral range of 50–3500 cm^−1^ at a resolution of 2 cm^−1^ using 532 nm laser excitation wavelengths, and X-ray photoelectron spectroscopy (XPS) was performed using an Electron Spectroscopy for Chemical Analysis II system (AXIS SUPRA, Kratos, UK) with a monochromatized aluminum K anode (350 W, 25 mA) at the National Center for Interuniversity Research Facilities (NCIRF) at Seoul National University. Atomic field microscopy (AFM) was used to show these characteristics, and the measurement conditions were as follows. The sample size was 5-μm diameter square, and analysis was conducted in contact mode using a cantilever with a resolution of 0.2 nm for the X/Y axis and 0.01 nm for the Z axis. The Brunauer–Emmett–Teller (BET) surface of the samples (100 mg) was determined from triplicate analysis using the BJH equation N_2_-desorption method (Quantachrome, PM33, USA). The contact angles were measured by dropping 20 μL of water droplets onto the sample surface. The relative amine number was calculated by calculating the molar amount of PM added in method parts. The morphological analysis of the CNT@CPM nanocomposite samples was performed using high-resolution transmission electron microscopy (HR-TEM; JEOL Ltd, JEM 3010, Japan) with a resolution of 0.17 nm and field emission scanning electron microscopy (FE-SEM; TESCAN, MIRA LMH microscope, Tescan, Czech Republic). The FE-SEM studies were conducted using samples that had been sputter-coated with a 5-nm Pt layer prior to analysis. Energy dispersive X-ray spectroscopy (EDS, BRUKER, Quantax, USA) and line-scan analysis were conducted under the resolution conditions of 125 eV and SDD type.

### Humidity sensing properties

A humidity and temperature controller (Model TM-NFM-L, Jeio Tech, Korea) was used to measure relative humidity (RH) at 25 °C. Resistance was measured with an LCR meter (Model EDC-1635, 0.1 Ω–20 MΩ) at 1 kHz, 1 V, and 25 °C. The resistance variation graph for humidity was analyzed every 10 s by measuring the resistance of each humidity every 10 s when the relative humidity was adjusted every 10 percent from 30%RH to 100%RH. The hysteresis curves were repeated 5 times from 30%RH to 100%RH and again to 30%RH, and dynamic transient response curves were measured by changing from 30%RH up to each RH. The long-term stability test of each CNT@CPM flexible sensor was conducted by measuring the resistance once every 3 d for over 1 month while sensor was maintained at 50, 70, and 90%RH. The hysteresis extracted the average of the relative humidity differences (i.e., the average of the x-axis differences) between the adsorption and desorption graph at the same resistance value in range of 30–100%RH [25]. These average values compared in steps of 10% RH for each sample.

### Electrochemical analysis

All electrochemical analyses were performed directly using the CNT@CPM flexible sensors. The current–voltage (I-V) curves were measured using a Voltage sag protector (VSP) potentiostat (BioLogic, France), the measurement conditions were increased and decreased by 50 mV per second from − 1.0 V to 1.0 V at 25 °C and 30%RH, and the average value was used after three repeated measurements. A three-electrode system was used with Ag/AgCl (3.0 M KCl) from Cypress systems (Lawrence, KS, USA) as a reference electrode, a 0.5 mm Pt wire counter electrode, and 4 mm diameter CNT@CPM flexible sensors as the working electrode. Cyclic voltammetry (CV) curves were measured in a mixed solution of 2.0 mM K_3_Fe(CN)_6_ (0.5 M KCl) and 2.0 mM K_4_Fe(CN)_6_ (0.5 M KCl). Impedance measurements (EIS) were performed after each sample in the impedance analyzer (PalmSens BV, Nederland) was for 30 min per humidity range in the 25 °C humidity chamber, and the measurement conditions were increased and decreased by 0.01 V per second from − 1.0 V to 1.0 V at frequencies (61 = 10/dec.).

### Statistical analysis

All experiments were repeated at least three times and data are presented as means ± standard deviation (S.D.).

## Results and Discussion

**Fig. 1:**
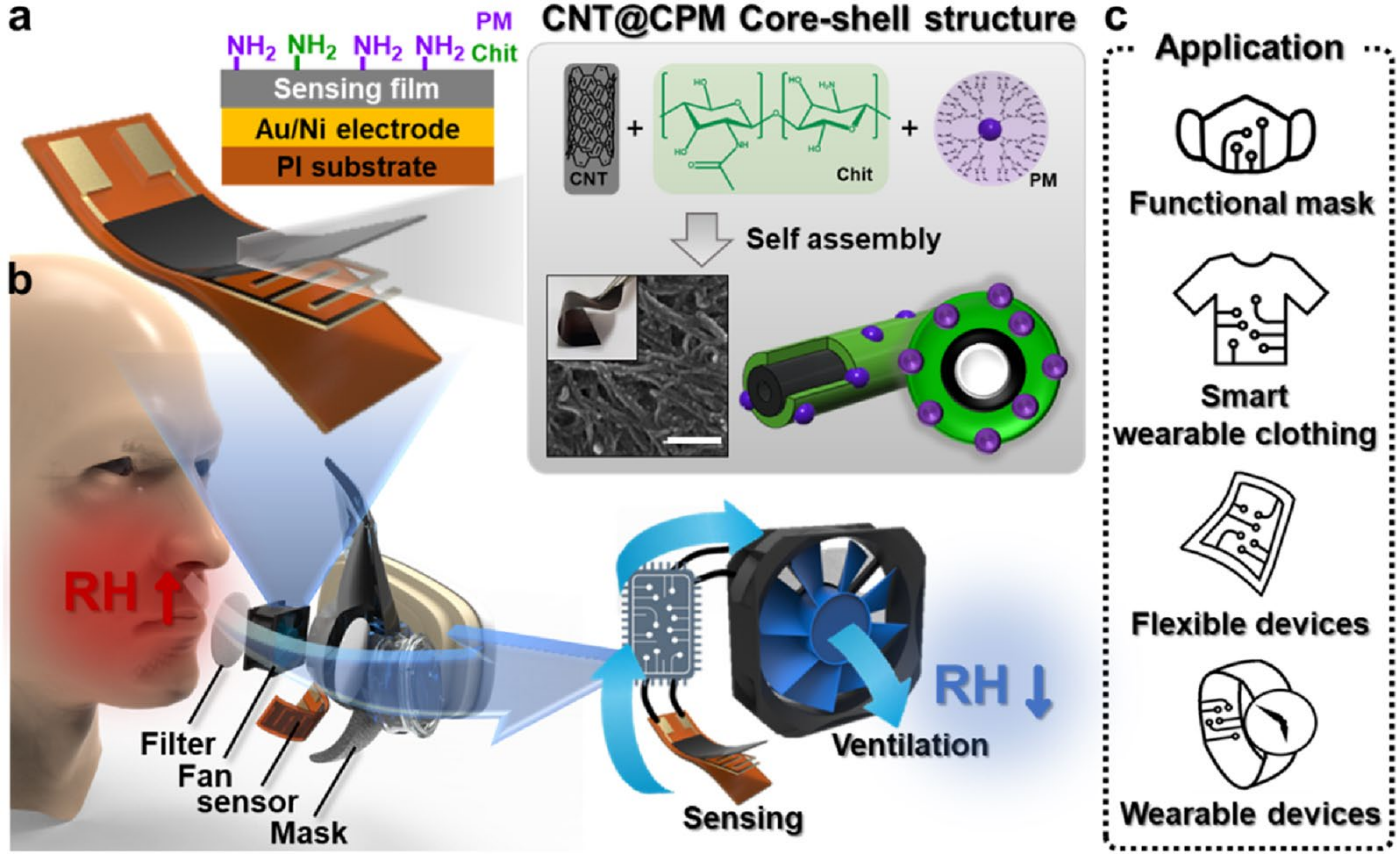
A schematic illustration summarizing the overall process. **a** Process for making the flexible humidity sensor based on a flexible CNT@CPM nanocomposite (CNT core, and Chit/PM shell) structure immobilized and physically adhered to an Au/Ni electrode on the PI flexible surface. **b** Schematic and composition plot for an actual application involving a respiration mask with a ventilation system. **c** Various types of commonly available smart wear

A schematic illustration summarizing the overall progress of our present paper is presented in Fig. 1. A flexible sensor based on the CNT@CPM nanocomposites was composed of a CNT core and PM trapped in a Chit-shell structure. As depicted in Fig. 1a, Chit acted as a biocompatible glue on the surface of CNT, an excellent conductive material, as the shell and PM was utilized to increase the humidity efficiency via the augmentation of amine groups that were formed as core–shell structures via self-assembly by adjusting the pH level. More specifically, PM was trapped in the shell of the Chit and immobilized, resulting in an increased surface area, and it had a direct effect on sensitivity and linearity for effective humidity sensing. It is worth noting that in this process, the core–shell fiber could be formed as a flexible nanocomposite film pursuant to strong hydrogen bonds. These advantages are reflected in the flexible humidity sensor for smart wear. The CNT@CPM composites were immobilized on a flexible polyimide (PI) substrate, which was made from an Au/Ni electrode through masking, producing a flexible humidity sensor. To monitor respiration, we fabricated a humidity-controllable mask by integrating a microcontroller that activated the fan when the humidity was above 70% and a high-performance CNT@CPM-3 humidity sensor. It was confirmed that not only did it monitor the respiratory rate, depth, and occurrence of exhalation or inhalation, but it also controlled the humidity level inside the mask, validating its performance for realistic applications (Fig. 1b). This flexible humidity sensor is not limited to masks and could be used for multiple smart wear in everyday life (Fig. 1c).

**Fig. 2:**
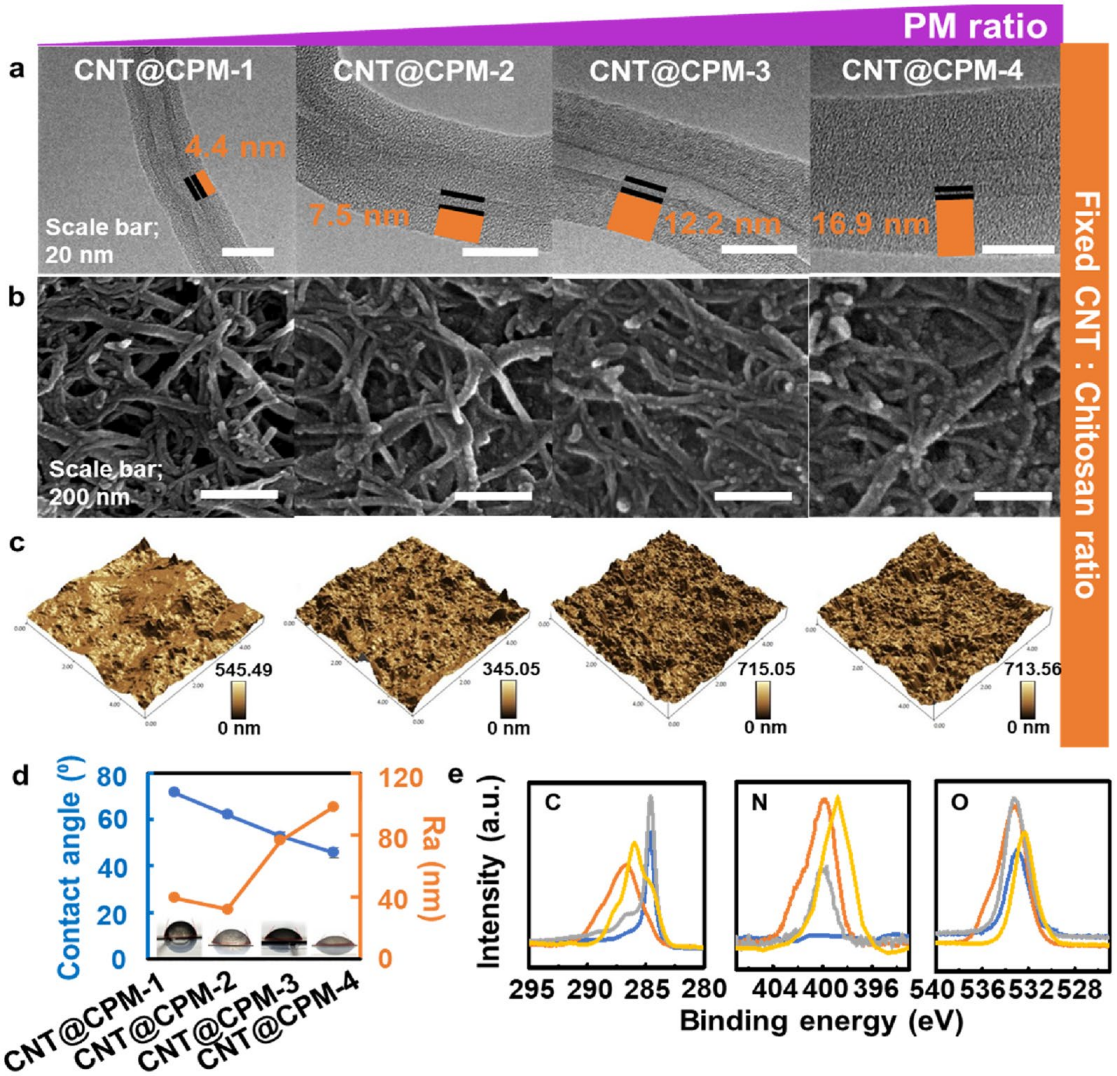
The morphology and physicochemical properties of nanocomposites formed at different PM concentrations. **a** HR-TEM images of shell thickness ranged from 4.4 to 17.4 nm depending on the PM ratio. **b** FE-SEM images of CNT@CPM; CPM shell thickness became thicker. **c** AFM surface images of CNT@CPM, exhibiting a highly rough nanoto-pographical surface, **d** with a roughness ranged from 32.1 nm to 98.2 nm as the ratio of PM increased, and contact angles; hydrophilicity varies depending on concentration of PM. **e** XPS scanned revealing that the self-assembled CNT@CPM had clear shift of binding energy

The morphological changes and properties of the samples formed at different PM concentrations (0–10 wt%) are compared in Fig. 2. Based on HR-TEM images (Fig. 2a), the CPM thickness values of 4.4 ± 0.51 nm for CNT@CPM-1, 7.5 ± 0.68 nm for CNT@CPM-2, 12.2 ± 0.94 nm for CNT@CPM-3, and 17.4 ± 1.77 nm for CNT@CPM-4, as derived from 50 thickness measurements. The thickness values used to acquire the average thickness are shown in the distribution graph in Additional file 1: Fig. S2. The HR-TEM images clearly show an increase in the thickness of the shell layer on the surface of CNTs with increasing concentration of PM, clearly indicating the formation of the CNT core and Chit/PM-shell structured nanocomposites. The PM was very evenly distributed among the polymers of Chit, resulting in an increase in the overall coating thickness as the shell structure became loose (Additional file 1: Fig. S3). These results are described with a consistent nano-rough topology. The FE-SEM images (Fig. 2b**)** also showed the same results as those of the HR-TEM. Obviously, the nanopores between the nanofiber strands shown in CNT@CPM-1 became thicker and were being blocked owing to the increasing PM ratio. Strong interaction with density changes according to fiber thickness can allow the flexible PI-based sensor to be strongly and reliably attached. The AFM images were measured to determine the change in the hydrophilic properties according to the ratio of PM. The AFM data (Fig. 2c, d) showed that the PM was very evenly attached to the core–shell structure of CNT@CPM-1. The surface morphological topography emerged as peaks and valleys originating from the structure of CNT@CPM. The surface roughness of each substrate, given by the average roughness parameter (Ra) value of 39.7 ± 1.24 nm for CNT@CPM-1, 32.18 ± 2.32 nm for CNT@CPM-2, 76.43 ± 4.11 nm for CNT@CPM-3, and 98.25 ± 6.83 nm for CNT@CPM-4, showing a threefold difference. The CNT@CPM nanocomposites well reflected that the nanocomposites exhibited a consistent morphology with varying shell thickness. The relative height deviation increases because the diameter of CNT@CPM increases according to the amount of PM (Additional file 1: Fig. S4). These innate hydrophilic properties of Chit and PM, as well as the highly nano-rough surface topological features of the nanocomposites, might influence the increased hydrophilicity [26]. The nanocomposites also exhibited a level of water-uptake capacity, which allowed for the rapid appearance of adhesion and reactivity with humidity. The wettability (decrease in the water contact angle, Fig. 2d) increased as the PM ratio increased (each contact angle results are shown in Additional file 1: Fig. S5). These phenomena were due to the increased ratio of primary amines caused by PM, which increased the surface roughness and hydrophilicity [27]. Qualitative analysis was conducted using XPS to confirm whether the CNT, Chit, and PM were efficiently combined with the core–shell structure. The XPS analysis is shown in Fig. 2e for comparison and analysis of the variation in the binding energy between elements by the efficient core–shell structure. The CNTs-related characteristic peaks at 284.68 (C 1s) and 533.08 eV (O 1s) and Chit-related characteristic peaks at 286.68 (C 1s), 533.28 (O 1s), and 399.98 eV (N 1s) were observed. The CNT@CPM-3 nanocomposites spectra of oxygen and nitrogen were slightly shifted (O 1s; 533.28 to 532.28 eV, and N 1s; 399.98 to 398.42 eV). After PM was added, the peaks related to Chit and CNT@CPM-3 were generally shifted to lower positions by 1.0–1.56 eV, indicating intimate physicochemical interactions between the highly polar functional groups of the PM and Chit (C = O, N–H, and O–H). These phenomena indicated efficient complexation due to the large contact area between the CNTs, Chit, and PM. Numerous hydrogen bonds were formed between the Chit shell and the PM functional groups in the core–shell structure, consequently decreasing their binding energy [28, 29] (full spectra in Additional file 1: Fig. S6). The characteristic peaks of CNTs, Chit, and PM [30, 31] are well represented in the FT-IR spectra (Additional file 1: Fig. S7) of the CNT@CPM-3 nanocomposite. The RAMAM analysis results (Additional file 1: Fig. S8) from CNTs, CNT@CPM-1, and CNT@CPM-3 all demonstrated feature D and G bands [32]. The RAMAN spectra of post-composition via the core–shell nanocomposite CNT@CPM-3 simply superimposes the relative strength change for the D and G bands, suggesting a change in the amorphous content of CNT@CPM-3. The I_D_/I_G_ ratio of the core–shell nanostructure increased from 0.839 to 1.196, as it was synthesized into Chit and PM.

These results were also confirmed using BET measurements of the surface area. The results of measuring the surface area according to the PM ratio of CNT@CPM are summarized in Table 2. As the ratio of PM increased, the surface area and pore volume increased; however, a tendency to decrease again occurred at 10 wt% PM. This appeared to be caused by reduced nanopores as hydrogen bonds became stronger, because the number of primary amines and coating thickness increased as more PM was embedded in Chit.

**Table 2:** Surface area, pore size, and pore volume measured by BET N_2_-adsorption method at each samples

	CNT@CPM-1	CNT@CPM-2	CNT@CPM-3	CNT@CPM-4
Surface area (m^2^ g^−1^)	11.09 ± 1.20	26.36 ± 4.93	25.14 ± 5.13	12.11 ± 3.56
Pore size (nm)	3.10 ± 0.16	3.80 ± 0.50	3.73 ± 0.77	3.76 ± 0.56
Pore volume (cc g^−1^)	0.05 ± 0.01	0.06 ± 0.01	0.06 ± 0.01	0.05 ± 0.02

**Fig. 3:**
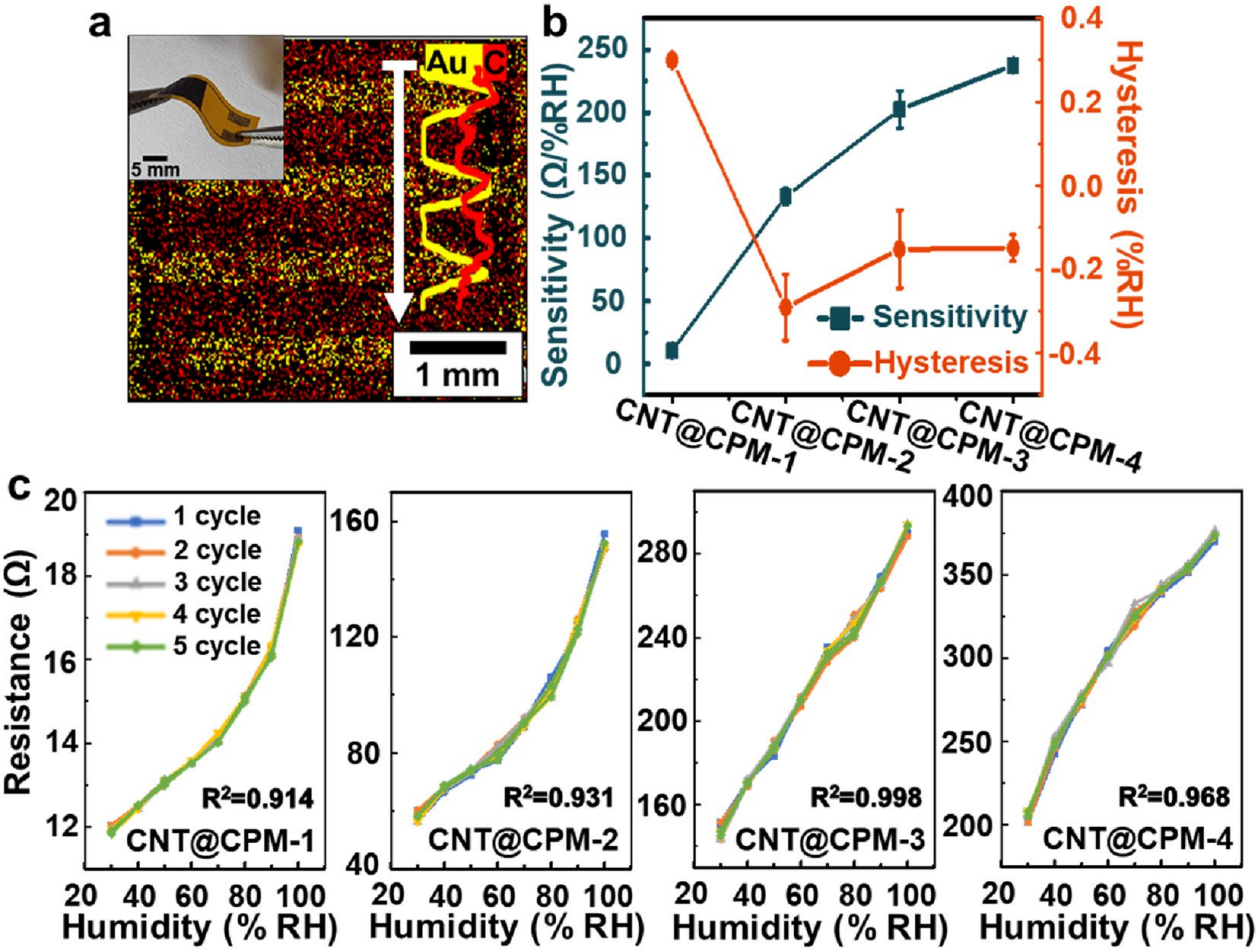
Physical adhesion of a flexible sensor with CNT@CPM and humidity dependence of different PM ratio. **a** Actual photograph of a humidity sensor and EDS mapping and line scanning analysis; the flexible sensor was evenly coated and revealed to be completely covered by individual CNT@CPM core–shell nanocomposites. **b** Sensitivity and hysteresis integrated graph, showing sensitivity was 10 to 237 Ω/%RH from 0 to 10 wt%, and hysteresis was (− 0.291 ± 0.079) ~ (0.300 ± 0.001) %. **c** Humidity hysteresis curves of CNT@CPM, showing high reproducibility, repeatability, and constant values; linearity reached 0.998 (R^2^) in CNT@CPM-3

The CNT@CPM nanocomposites were immobilized, as shown in Fig. 3a, on a sensor physically adhered to the Au/Ni electrode on the PI flexible surface, and it can be seen that it was still flexible owing to the efficient structure of the core shell after immobilization (applied using flexible properties of CNT@CPM film). As the conductive sensing film was immobilized, the gold electrode was connected to each other, and the primary amines were greatly increased by the added PM (over 40 times to 132.7 times, Table 1), which increased the reaction and efficiency of water molecules in humidity. A photograph and actual size of a bare flexible sensor is shown in Additional file 1: Fig. S9. EDS mapping and line scanning analysis were conducted to confirm that the materials were evenly coated on the sensor. By showing the distribution of C and Au in the EDS mapping and line scanning images of the humidity sensors, it was shown that gold electrodes were fully covered by individual CNT@CPM core–shell nanocomposites (the FE-SEM images for each ratio of PM are compared in Fig. 2b, all clearly showing core–shell structures). The EDS results (Additional file 1: Fig. S10) and mapping images (Additional file 1: Fig. S11) clearly show that these were evenly coated. Additionally, the FE-SEM image of the cross-sectional flexible humidity sensor (Additional file 1: Fig. S12) shows that CNT@CPM-3 is coated very stably, and thickness of each layer can be confirmed that thickness of Au and Ni are average 3.75 ± 0.04 μm and 1.80 ± 0.01 μm, respectively, and coated CNT@CPM layer is average 2.76 ± 0.16 μm. The humidity sensor showed that the even coating of these core–shell structures not only provides a wide surface area for responsiveness to humidity, but it can also establish a fast and consistent response. The CNT@CPM contained numerous amino groups (− NH_2_) that can act as proton donors and acceptors, providing a possible proton transport mechanism with humidity [33]. Here, the electrical conductivity could be generated by H^+^ ions generated through the dissociation of humidity molecules [34] that were first chemically adsorbed on the amine surface and increased by the transfer of the generated H^+^ ions through the humidity phase that is physiosorbed, as shown in Additional file 1: Fig. S13 (Grotthuss chain reaction) [34, 35]. Consequently, a higher level of humidity would increase the number of physically adsorbed humidity molecule layers, allowing faster proton hopping among adjacent water molecules in a continuous water layer and increasing the resistance [34]. The reason for the increase in the electrical resistance of CNTs, due to adsorption of water molecules, can be the donation of electrons by adsorbed water molecules to the valence band of CNTs, which exhibit a hole transport similar to that of a *p*-type semiconductor [21]. Electron transfer quickly reduces the concentration of holes in the CNTs, resulting in a rapid increase in resistance. This implies a decrease in the number of holes and an increase in the separation between the Fermi level and valence band [36]. The resistance variation curves of the CNT@CPM nanocomposite humidity sensors exhibited a linear function of RH with a positive slope (dR/dRH > 0). Here, the increasing number of water molecules with an extremely high resistance compared to the CNT@CPM phase caused a positive slope [34]. The response and sensitivity (*S*) values of the CNT@CPM nanocomposite sensors to humidity were calculated using the following equations [34]:

1 $${\text{Response }} ( R) = \Delta R$$

2 $${\text{Sensitivity }} ( S ) =\Delta R/R_{O} /\Delta {{\% }}RH$$

where ΔR/R_0_ = (R_RH_ − R_0_)/R_0_ is the resistance change, R_0_ is the initial resistance value (30 RH%) of the device, and R_RH_ is the steady-state resistance when exposed to different RH values. The sensor response, curve slope, and linearity of the sensors resulting from the sensor materials with different PM concentrations are shown and summarized in Fig. 3b, c, and Additional file 1: Table S1. The CNT@CPM nanocomposites exhibited high sensitivity and low hysteresis (Fig. 3b). Sensitivity and hysteresis integrated graph, showing sensitivity was 10 to 237 Ω/%RH from 0 to 10 wt%, and hysteresis was (− 0.291 ± 0.079) ~ (0.300 ± 0.001) %. Furthermore, the curve showing hysteresis through repeated testing of sensors (even 5 cycles) according to each humidity showed high reproducibility, repeatability, and low measuring factors (Fig. 3c). These data clearly show that the CNT@CPM-3 nanocomposite sensors have relatively high values of sensor response, sensitivity, and linearity among the tested sensor materials. When the PM concentration ratio increased from 0 to 5 wt%, the linearity of the resistance curves gradually increased, reaching an R^2^ value of 0.998 (CNT@CPM-3), because the increase in PM concentration caused an improvement in the sensor response by efficient hydrogen bonding due to an efficient reaction even at low humidity. At 10 wt%, the R^2^ value was reduced to 0.968 because the reactivity was too high in the low humidity range. EIS measurements for our sensors according to humidity showed that %RH was related to the direct resistivity of the sensor itself (Additional file 1: Fig. S14). Nevertheless, the resultant absolute impedance Nyquist plots disclosed no useful information and did not follow the common EIS curves. These plots only indicated that there were differential charge transfers according to the amounts of water molecules in chamber. Higher water molecule content in the cell could increase the charge transfer resistance and the gap between the carbon electrodes, eventually leading to the resistance trends observed in Fig. 3c. As the humidity increased from 30 to 100%, the resistance of the CNT@CPM-3 sensors increased from 150 to 300 Ω. In addition, there is a rapid and direct increase in the resistant responses when the humidity increases from 30 to 100% RH, as shown in the logarithmic representation shown in Additional file 1: Fig. S15, which indicates that moisture absorption is enhanced in these humidity sensors. These results demonstrate that the resistance exhibits excellent logarithmic linearity toward RH as an excellent humidity sensor [37]. Additional file 1: Fig. S16 shows the I-V characteristics of the sensors under different PM concentrations at 25 °C. It is clear from these I-V curves that the current of the sensor decreases with an increase in the core–shell structured CNT@CPM nanocomposites. The curves are linear, proving the ohmic characteristics of the contacts, as the resistance of the sensor increases with the increase in PM nanocomposites. Hence, the CNT@CPM-3 nanocomposite could be used as the maximum concentration to maintain these I-V characteristics. Figure 3c shows that the resistance of the sensors increases significantly with an increase in humidity, which is consistent with the CV curve shown in Additional file 1: Fig. S17, resulting in the stable maintenance of the resistance values of the humidity sensor. CV data indicates that the oxidation and reduction peaks of CNT@CPM nanocomposites are well shown and that compared with those of CNT@CPM-1, the peaks of CNT@CPM-3 modified electrodes increased dramatically according to the increased electrochemical reactions between the electrolytes and the amino groups of CNT@CPM nanocomposites on the electrode surface [38].

**Fig. 4:**
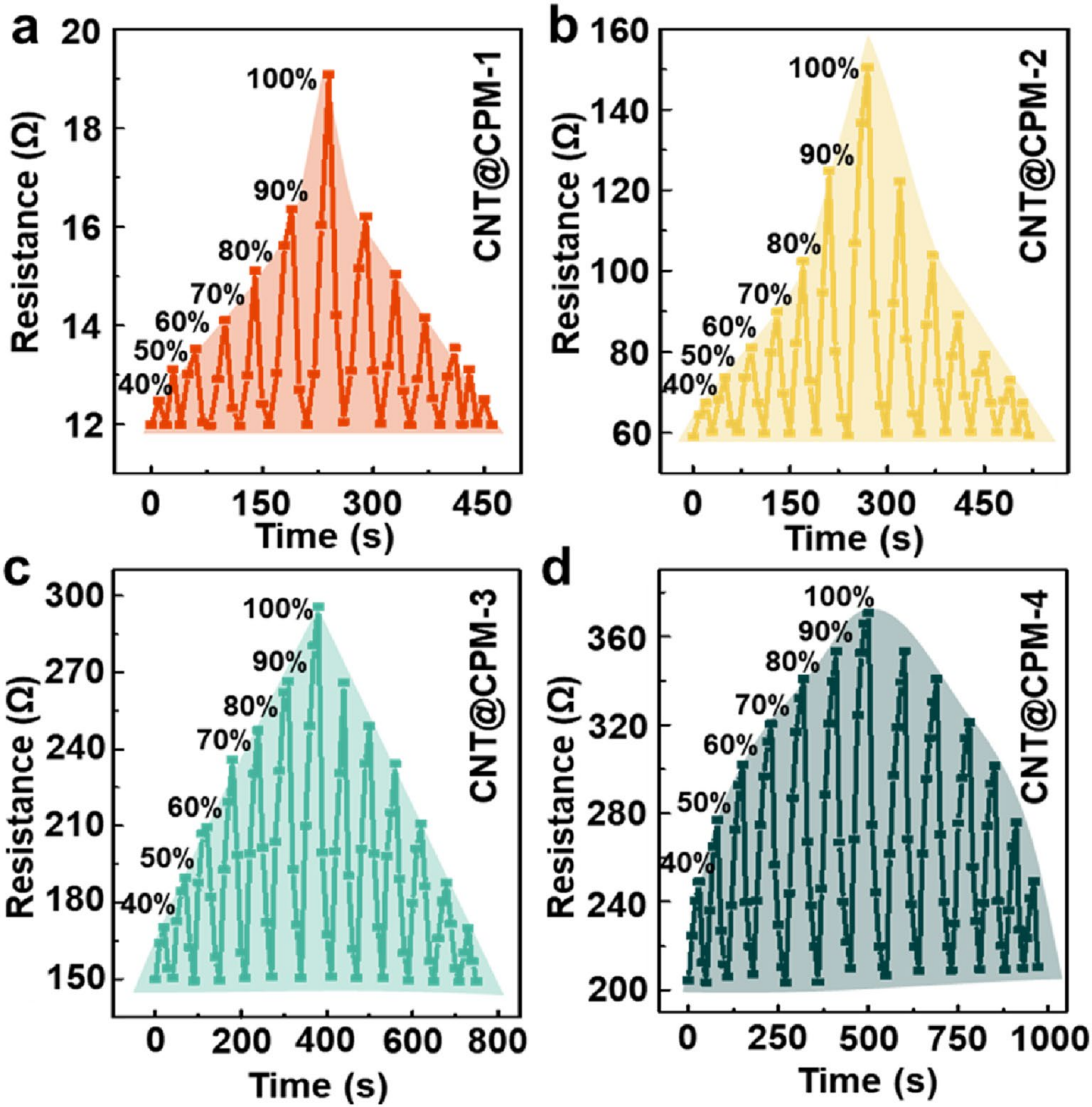
Dynamic transient response of flexible CNT@CPM sensors. **a** CNT@CPM-1, **b** CNT@CPM-2, **c** CNT@CPM-3, and **d** CNT@CPM-4, showing patterns were exponential (0 and 3 wt% PM), linear (5 wt% PM), and logarithmic pattern (10 wt% PM) with less than 10–40 s of response time

The dynamic transient responses were among the most important characteristics for evaluating the performance of the humidity sensors (Fig. 4). The patterns differed depending on the PM ratio. The sensors containing small amounts of PM (CNT@CPM-1; Fig. 4a, and CNT@CPM-2; Fig. 4b) exhibited an exponential trend in the sensitivity graph. When the PM ratio was relatively high, the graph showed a linear trend for CNT@CPM-3 (Fig. 4c) and a logarithmic trend for CNT@CPM-4 (Fig. 4d). These changes in the trends were due to the rapid response to resistance changes in the low humidity range caused by a hydrophilicity that increased with the increasing number of free amines from the added PM. Nevertheless, the response time of the humidity sensor was still shown to change very quickly, with less than 10–40 s, when RH was increased or decreased from 30%RH to each %RH. These characteristics enable efficient adsorption and desorption from water molecules due to influence of a large surface area by CNT@CPM core shell structure. After aggregating these results, we applied the CNT@CPM-3 flexible sensor, which has the most linear responsiveness, for subsequent experiments.

**Fig. 5:**
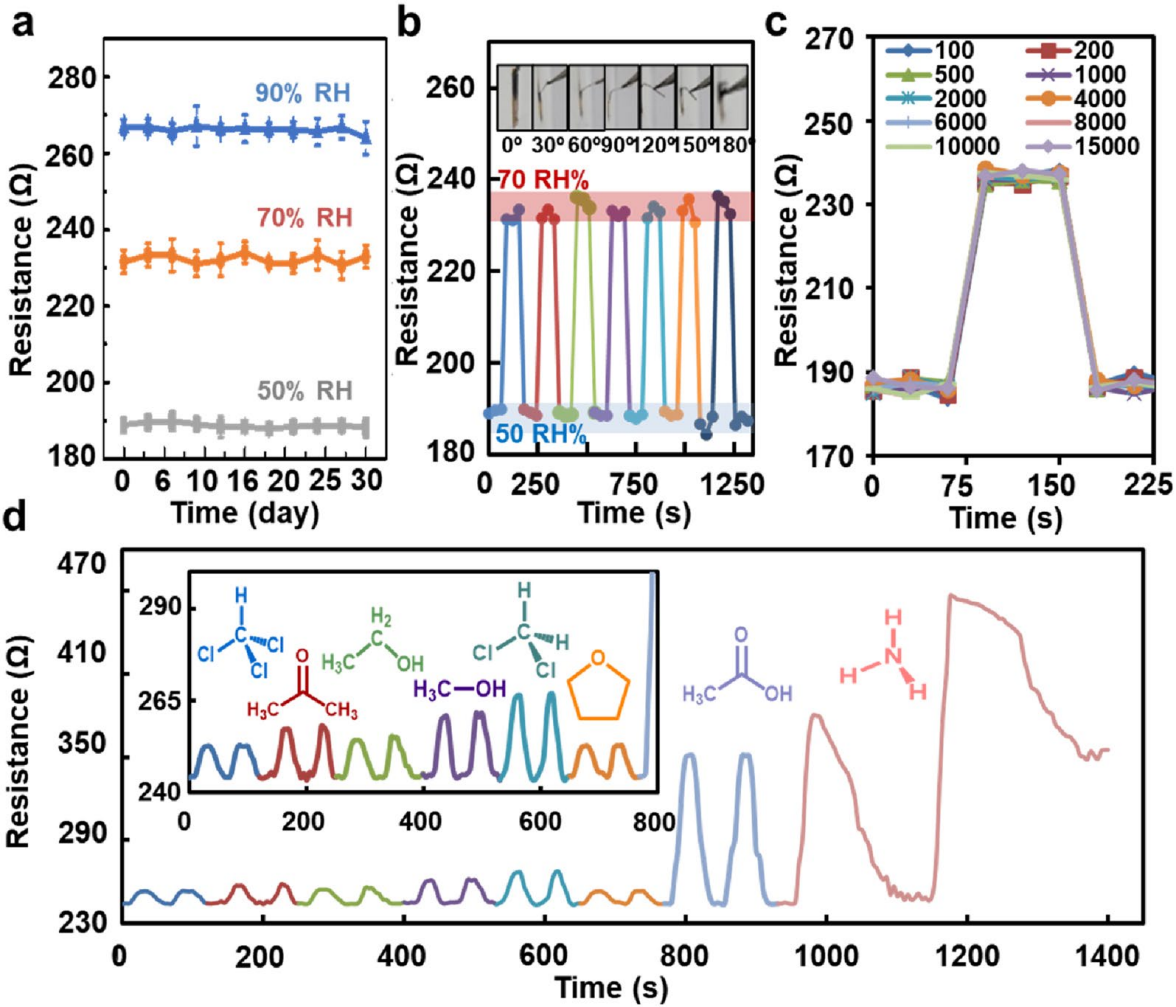
Mechanical stability property of CNT@CPM-3. **a** Long-term stability, showing sensor maintained consistent resistance values at 50, 70, and 90 RH% humidity for 30 days. **b** Stability at each bending angle, showing sensor maintained consistent responsiveness from all angles. **c** Mechanical durability test of sensor under repeated bending cycles, showing stable resistance values after 15,000 tests. **d** Detection of several organic vapors; several different polar organic gases can be detected

Our sensor could provide fast and stable attainment of sensitivity and long-term stability as an optimal humidity sensor. The CNT@CPM sensors were immobilized on a flexible sensor that could provide good contact with the skin or wearable devices. However, the development of wearable electronics requires long-term and mechanical stability characteristics that require sensor reliability and durability under harsh conditions. The long-term stability results of CNT@CPM-3 showed few resistance changes for each relative humidity over a period of over 1 month (Fig. 5a), indicating that it remains a stable sensor over a long duration at 50, 70, and 90%RH. Different proportions of sensors (CNT@CPM) showed the same long-term stability results and unchanged morphologies (Additional file 1: Fig. S18, S19). The mechanical properties of the flexible sensor were evaluated by monitoring their responses under different bending angles (Fig. 5b). The resistance of the device did not change even after bending at 180°. Additionally, there was no change in resistance after 15,000 bending cycles, demonstrating the superior flexibility and durability of the sensor (Fig. 5c, Additional file 1: Fig. S19, S20). The RH response to humidity changes was evaluated over 15,000 bending cycles as well. These results demonstrated that the response was nearly unchanged even when the device was bent up to 180° over 15,000 bending cycles, which indicates the excellent mechanical durability, stability, and robustness of the CNT@CPM based humidity sensor. When bending was performed about 18,000 times, CNT@CPM was detached, which is a result of bending in a harsher condition than the actual condition. Therefore, the sensor can be effectively applied to practical applications such as skin-attachable or wearable devices. It is clear that the humidity detection performance of the flexible sensors remained almost constant within experimental errors at different bending numbers and angles, demonstrating their potential as a promising flexible wearable sensing device [39]. More interestingly, the sensor can also respond to other volatile polar organic molecules (chloroform, acetone, ethanol, methanol, dichloromethane, tetrahydrofuran, acetic acid, and ammonia) through resistance changes in a different manner, indicating its great potential for application in organic molecule sensing (Fig. 5d).

The humidity sensing performance and characteristics of the CNT@CPM flexible sensors were compared with other previously published resistance-based humidity sensor papers [40–50]. Obtained using various sensor materials, as shown in Table 3. The references showed that sensors with high sensitivity typically exhibited consistent performance due to high sensitivity and linearity but showed limitations, such as high response and recovery times, very high resistance ranges, and high-power requirements (low efficiency). Conversely, references showed that sensors with high response and recovery times can operate at low power (high efficiency) owing to low resistance but have limited operational range and inconsistent limitations owing to low sensitivity and linearity. For CNT@CPM flexible sensors, we demonstrated the advantages of having high sensitivity and linearity over a full range that complements the limitations of other existing references, consistent performance with low power (high efficiency), and high response and recovery times.

**Table 3:** Summary and comparison of flexible humidity sensing performance using various sensor materials

Material	Working range (%)	Working resistance (Ω)	Response time (s)	Recovery time (s)	Sensitivity (%)	Reference
CuO nano wire	20–80	0.5 ~ 3*10^6^	120	120	-	[40]
SnO_2_ nanowire	30–85	8*10^5^ ~ 2*10^7^	120–170	30–60	35	[41]
GO/PDDA film	11–97	0.5 ~ 2.7*10^6^	108–147	94–133	37.43	[42]
Black phosphorous	11.3–97.3	0.6 ~ 2*10^4^	255–360	10–480	42.85–99.17	[43]
VS_2_	0–50	5*10^3^ ~ 3*10^5^	30–40	12–50	300	[44]
Epoxy/CNT	10–90	0.8 ~ 1.3*10^3^	38	60	1,990	[45]
CNT/PVA fiber	46–98	> 0.8*10^4^	–	–	140,000	[46]
GO/PANI	11–97	–	8	5	20	[47]
Pt/GO fiber	6.1–99	> 500	2	4	4.51	[48]
GO/PEI	11–97	–	34	10	27.3	[49]
CNT/GO	10–80	13 ~ 96	4	3	9.8	[50]
CNT@CPM	30–100	12 ~ 370	10–40	10–40	56.7–111.1	This work

**Fig. 6:**
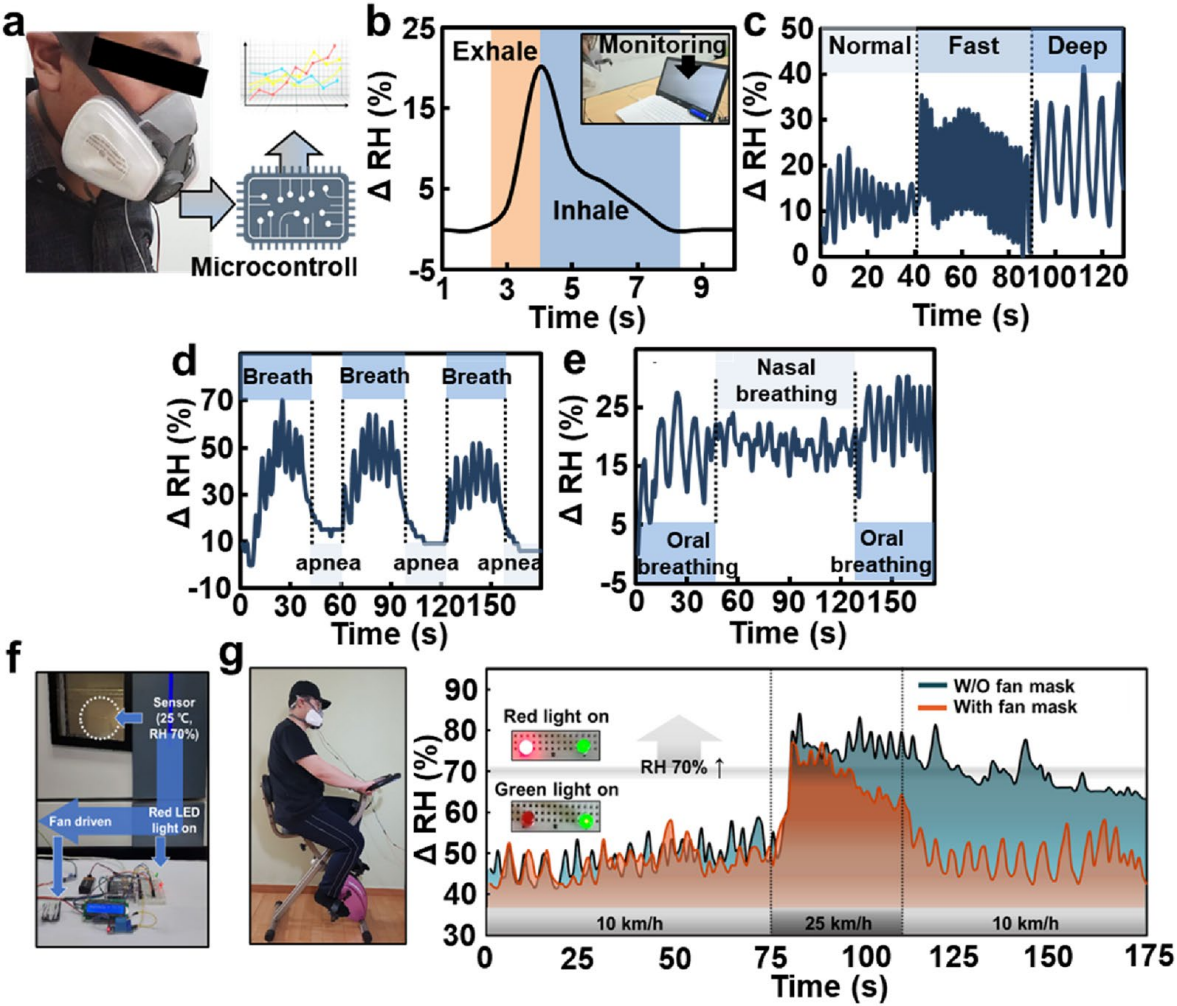
Performance evaluation of mask with micro-controlled flexible humidity sensor (CNT@CPM-3) for respiration measurements. **a** Photograph of a volunteer wearing respirator mask with the sensor fixed to monitor respiration. **b** Real-time respiration measurements of deep breathing. **c** Response of signal variations for different respiration. **d** Variation in relative response over time during respiration and voluntary apnea. **e** Detection of the rate and strength of an adult breathing via the mouth and nose (**f**) Photograph of the wearable device and actual operation for respiratory monitoring subject (**g**) Real-time respiratory monitoring results of a volunteer during cycling; Compared according to the intensity of exercise using ventilation system. (10 m∙s^-1^; mild condition → up to 25 m∙s^-1^; harsh condition → 10 m∙s^-1^; mild condition)

Fig. 6 shows the results of adsorption and desorption over real-time and photographs of the attached 3 M mask (6200 dust mask) for electrical signal amplification and sensor processing. Fig. 6a shows an image of the mask that was created by stitching a microcontroller and CNT@CPM-3 flexible sensor directly to an ordinary 3 M mask for real-time respiratory monitoring. The analog value of the CNT@CPM-3 flexible sensor was measured consistently as the resistance; therefore, a standard curve was drawn (Additional file 1: Fig. S21). This linear standard curve enabled us to produce accurate and consistent results. When deep breathing out, the exhaled air reached the humidity sensor, and the RH increased immediately, thus generating an exhalation signal. In contrast, during inhalation, dry air reaches the sensor, and an inhalation signal was generated (Fig. 6b). The actual performance of the sensor is shown in the Additional file 2: Video S1. With this high responsiveness and consistency, normal, fast, and deep breathing could be easily distinguished by the curve of the graph according to the frequency of rapid change in values, which presents a clear difference in current frequency (Fig. 6c). Apnea was simulated during the breathing process and measured to evaluate the performance of the sensor (Fig. 6d). The sensor accurately determined and measured repeated breathing and apnea, indicating that the sensor could be used to detect sleep apnea symptoms. In addition, the CNT@CPM-3 flexible sensor also distinguished breathing through the mouth and nose (Fig. 6e). The two types of breathing have different slopes of moisture exhaled due to the different levels of moisture exhaled from the mouth and nose. These results show that our humidity sensors have high sensitivity and rapid response, which can be used practically to accurately and conveniently monitor human respiration and other respiratory processes. The CNT@CPM-3 flexible sensor with fast response, low power, and consistency can be used as an efficient respiratory monitoring system. Fig. 6f shows that the humidity inside the mask can be adjusted through a ventilation system by applying additional fans to the previously identified mask humidity sensors. The humidity sensor was attached to the inside of the mask to measure humidity in real time and was configured to ventilate through a fan if it exceeded a certain humidity (set to over 70 RH%). To verify that the established system was working, the sensor was inserted into the preset humidity and temperature controller to confirm that the Light emitting diode (LED) lamp was lit by humidity and the fan was turning. The actual performance is shown in Fig. 6f, Additional file 1: S22 and Additional file 3: Video S2. Compared to the conditions without the fan system and with the fan system (Fig. 6g), the humidity gradually increased under medium-intensity conditions. Under harsh conditions, the humidity inside the modalities of the two masks increased rapidly, and the humidity inside was reduced quickly and effectively by the fan system. As a result, if the humidity inside the masks and smart clothes increased due to exercise and activities, it could be detected by sensors and ventilated through fans to create a pleasant internal environment. This fast, consistent, and responsive sensor and reactive fan ventilation system can be applied to masks and clothing, giving the wearer comfort in a partially or completely enclosed environment.

## Conclusions

Here we reported that resistance-type CNT@CPM flexible humidity sensors were realized from core–shell structure. The structure of the CNT@CPM nanocomposites was confirmed through qualitative and quantitative analyses. As the ratio of PM increased, the resistance graph over a RH range was transformed from an exponential graph to a logarithmic graph as the shell thickness and hydrophilicity increased. Among them, the CNT@CPM-3 flexible sensor showed a linear graph, and the sensor response hysteresis was only (− 0.152 ± 0.093) in the RH range of 30–100%, which was superior to conventional electric resistance humidity sensors. Moreover, we apply the sensor to smart-wear that could perform accurate human health monitoring of multiple types of respiration (normal, fast, deep, oral, nasal, and apneic breathing). These flexible sensors not only exhibited the ability to detect several organic gases, but it also showed consistent and stable results even after prolonged exposure to physical stress, such as bending. Taken together, novel CNT@CPM flexible sensor, which demonstrated stability and high and accurate response times, is a promising and convenient human healthcare monitoring system.

## Supplementary Information

**Additional file 1. **Information on the characterization of CNT@CPM nanocomposites and the flexible sensor coated with CNT@CPM, including shell thickness distribution graph, schematic illustration, water contact angle results, XPS test, FT-IR spectra, Raman spectra, EDS results, humidity sensor traits table, EIS curves, I-V characteristics, CV curves, long-term stability results and actual performance videos of humidity sensing.

**Additional file 2. **Video S1, The practical and actual application video of humidity sensor.

**Additional file 3. **Video S2, Actual performance video of humidity sensing and automatic ventilation system.

## Data Availability

The datasets used and/or analyzed during the current study are available from the corresponding author, upon reasonable request.

## Abbreviations

AFM: Atomic field microscopy
BET: Brunauer–Emmett–Teller
Chit: Chitosan
CNTs: Carbon nanotubes
PM: PAMAM dendrimer ethylenediamine core, generation 3
CNT@CPM: PM-embedded Chit/CNT nanocomposites
CV: Cyclic voltammetry
EDS: Energy dispersive X-ray spectroscopy
EIS: Impedance measurements
FE-SEM: Field emission scanning electron microscopy
FT-IR: Fourier transform infrared spectroscopy
HR-TEM: High-resolution transmission electron microscopy
I-V curves: Current–voltage curves
PI: Polyimide
RH: Relative humidity
XPS: X-ray photoelectron spectroscopy;
I_D_/I_G_: Intensity of D band/Intensity of G band
