# Supplementary material for: Wearable CNTs-based humidity sensors with high sensitivity and flexibility for real-time multiple respiratory monitoring
Source: Nano Converg. 2022 Aug 1;9:35. doi: 10.1186/s40580-022-00326-6 (PMC9343523; doi:10.1186/s40580-022-00326-6)
Supplement: Supplementary file 1 — Additional file 1. Information on the characterization of CNT@CPM nanocomposites and the flexible sensor coated with CNT@CPM, including shell thickness distribution graph, schematic illustration, water contact angle results, XPS test, FT-IR spectra, Raman spectra, EDS results, humidity sensor traits table, EIS curves, I-V characteristics, CV curves, long-term stability results and actual performance videos of humidity sensing. [file 40580_2022_326_MOESM1_ESM.docx]

Supporting Information

**Wearable nanocarbon-based humidity sensors with high sensitivity and flexibility for real-time multiple respiratory monitoring**

*Han-Sem Kim^a,1^, Ji-Hye Kang^a,b,1^, Ji-Young Hwang^c^, Ueon Sang Shin^a,b^**


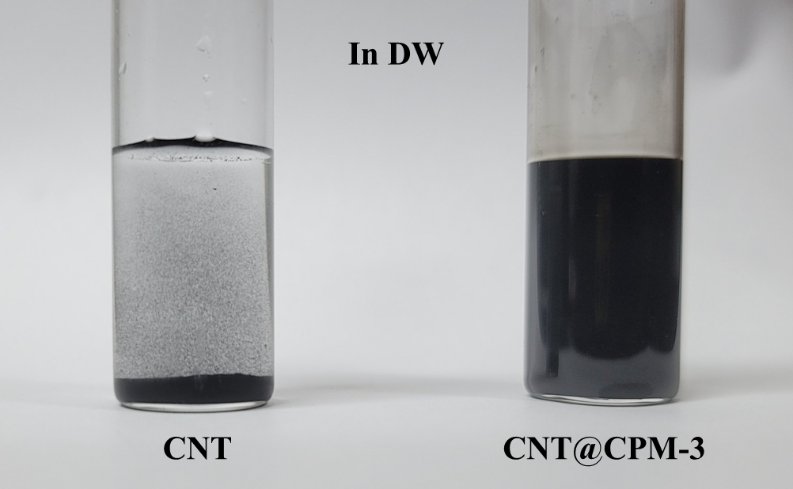


Figure S1. A photo of the dispersion stability of CNT@CPM.


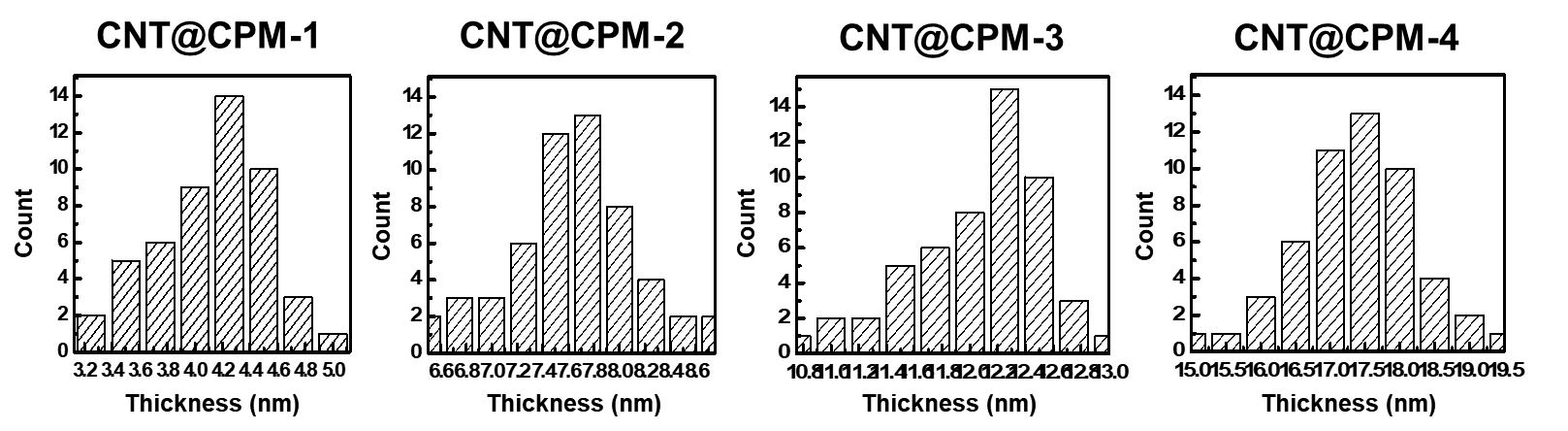


Figure S2. Shell thickness distribution graph of CNT@CPM.


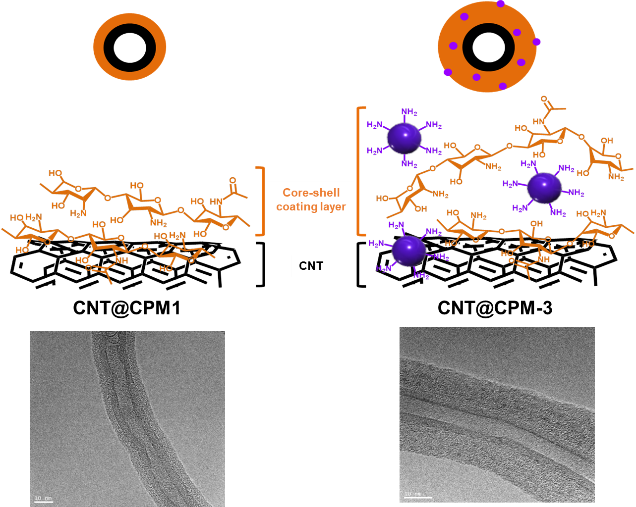


Figure S3. Schematic illustration of CS-MWCNT with PAMAM G3 nanohybrid structure.


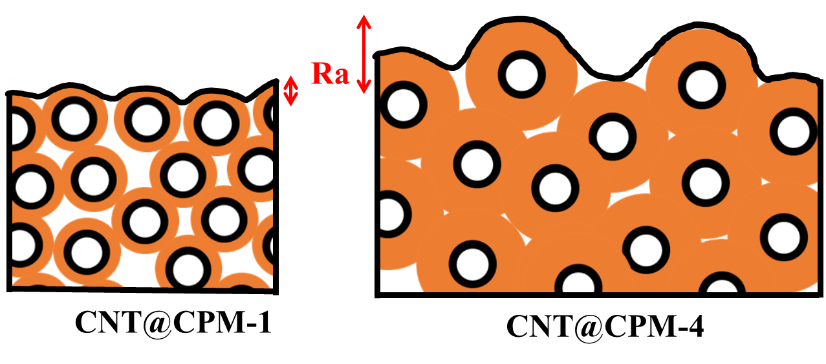


Figure S4. Schematic illustration of the cause of the difference in thickness depending on the CNT@CPM.


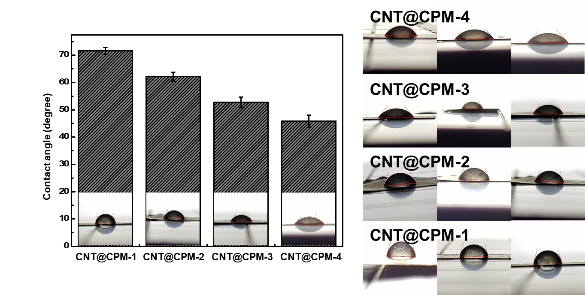


Figure S5. Water contact angle results of CNT@CPM.


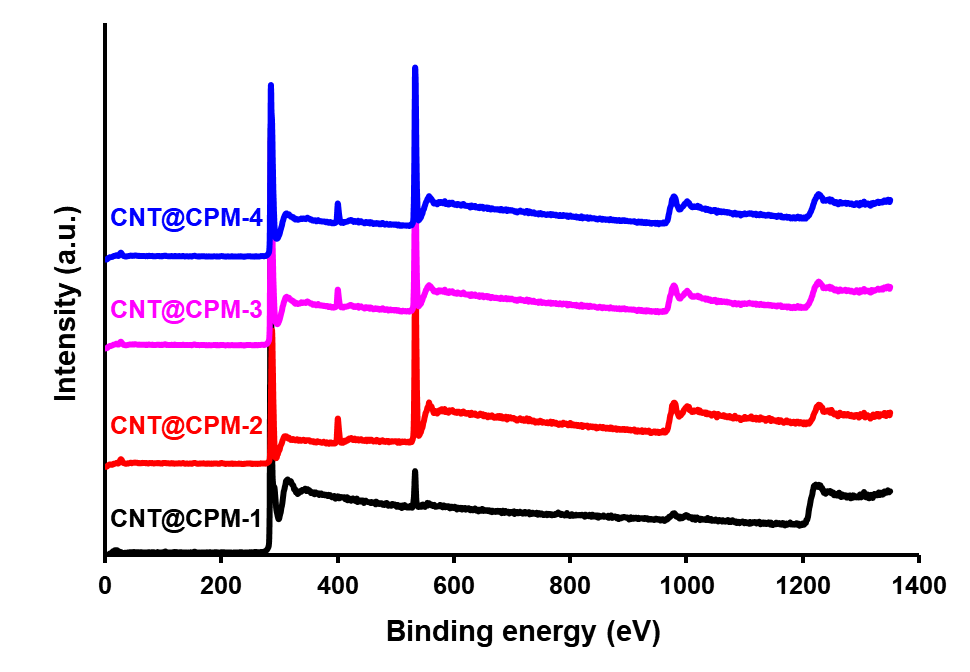


Figure S6. XPS full spectra of CNT@CPM.


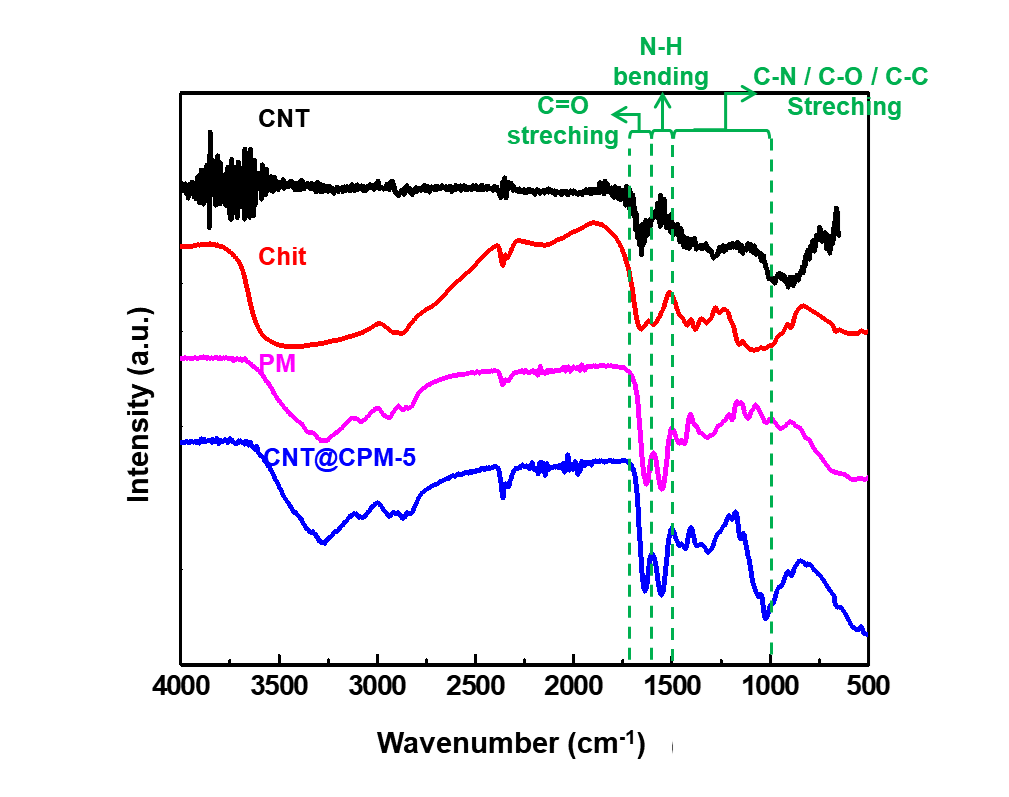


Figure S7. FTIR spectra of CNT, Chit, PM, and CNT@CPM-5.


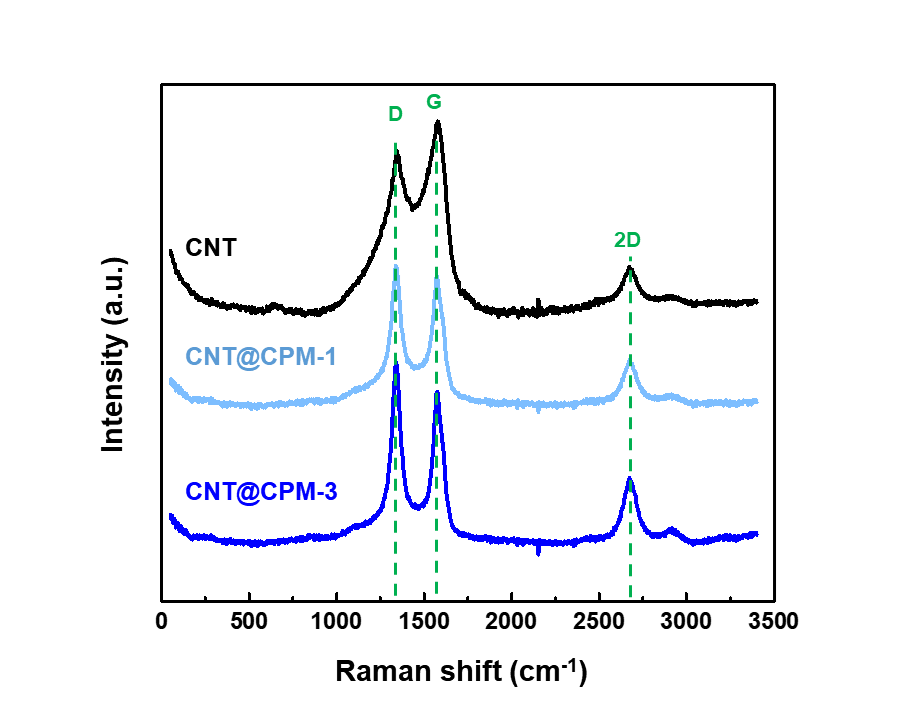


Figure S8. Raman spectra of CNT, CNT@CPM-1, and CNT@CPM-3.


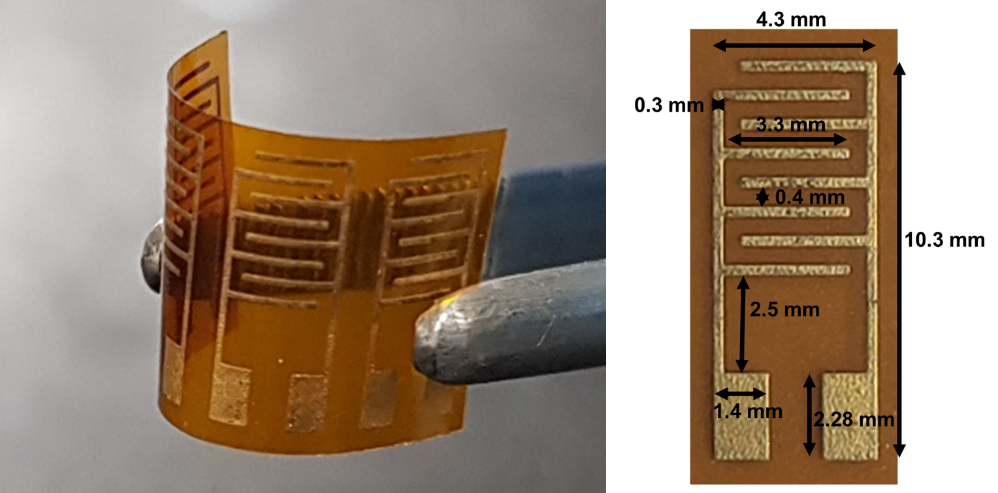


Figure S9. Photograph of bare flexible PI substrate sensor.


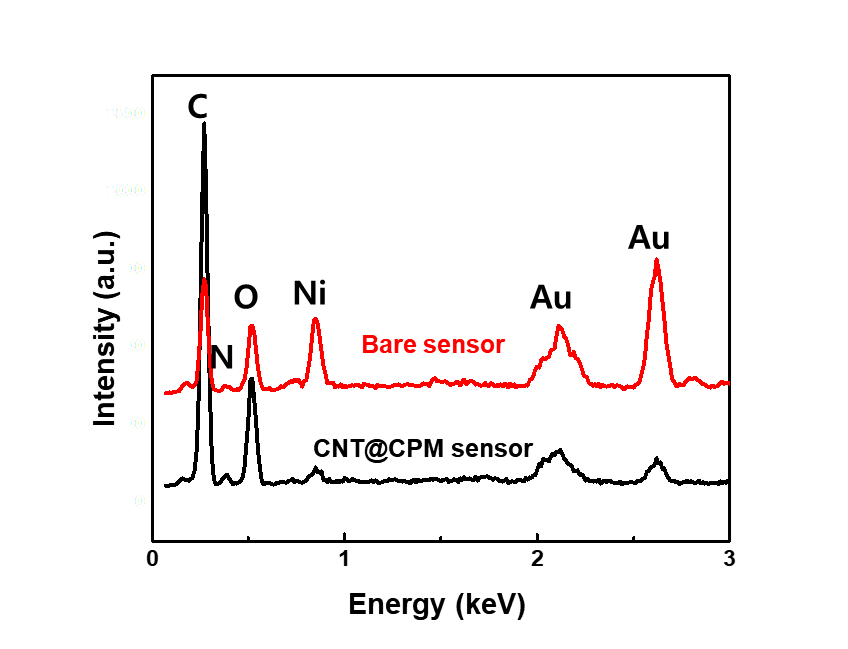


Figure S10. EDS results of bare sensor and CNT@CPM coated flexible sensor.


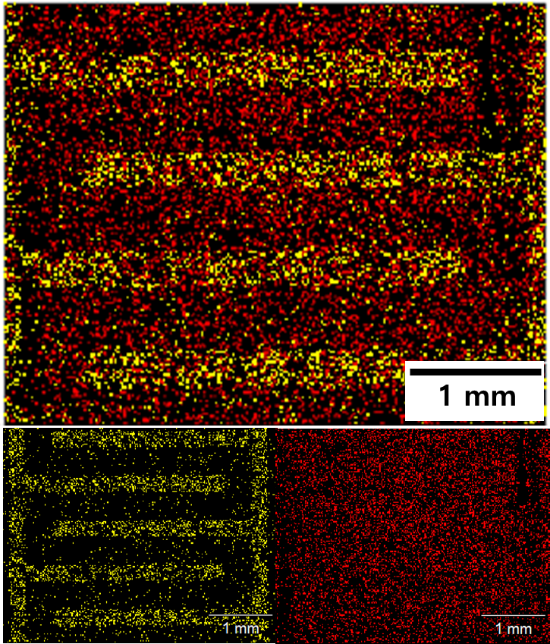


Figure S11. EDS mapping images of CNT@CPM flexible sensor.


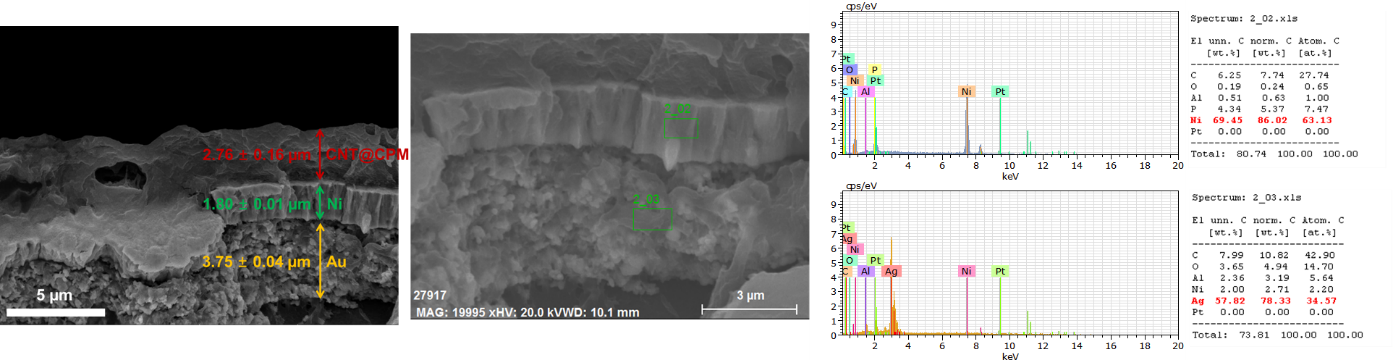


Figure S12. SEM image of cross-sectional flexible humidity sensor and each EDS data of Nickel and Gold electrodes layer (n=5).

Table S1. The sensor response, sensitivity, linearity, and hysteresis of CNT@CPM.

| Name | Sensor response (∆Ω) | Sensitivity (Ω/%RH) | Linearity (R^2^) | Hysteresis (%RH) |
| --- | --- | --- | --- | --- |
| CNT@CPM-1 | 7.247 ± 0.09 | 10.353 ± 0.11 | 0.931 | 0.3 ± 0.001 |
| CNT@CPM-2 | 93.168 ± 4.378 | 133.1 ± 6.254 | 0.914 | -0.291 ± 0.079 |
| CNT@CPM-3 | 141.62 ± 10.403 | 202.32 ± 14.861 | 0.998 | -0.152 ± 0.093 |
| CNT@CPM-4 | 166.34 ± 3.508 | 237.64 ± 5.011 | 0.968 | -0.149 ± 0.032 |

Sensor response = ∆Ω

Sensitivity = ${\Delta R}/{R_{O}/\Delta\%RH}$


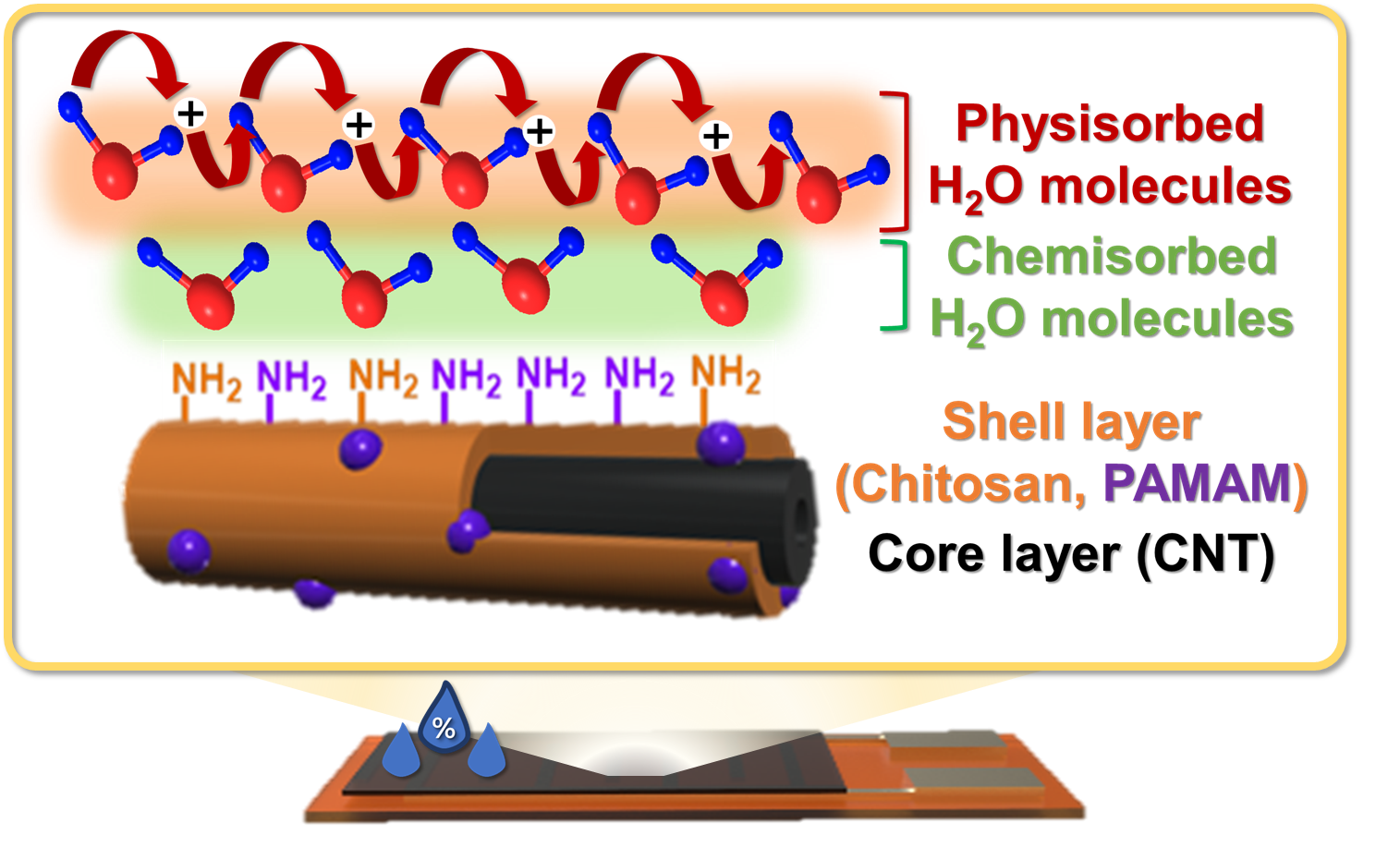


Figure S13. Schematic of flexible humidity sensing mechanism through CNT@CPM under humidity conditions; amino groups (Chit and PM) can act as proton donors and acceptors, providing a possible proton transport mechanism.


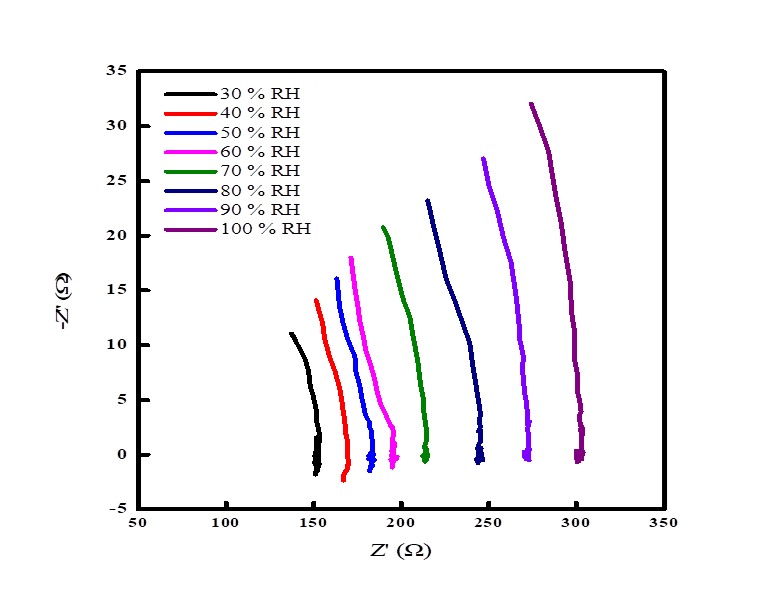


Figure S14. EIS curves of CNT@CPM-3, when the humidity increases from 30 to 100% RH.


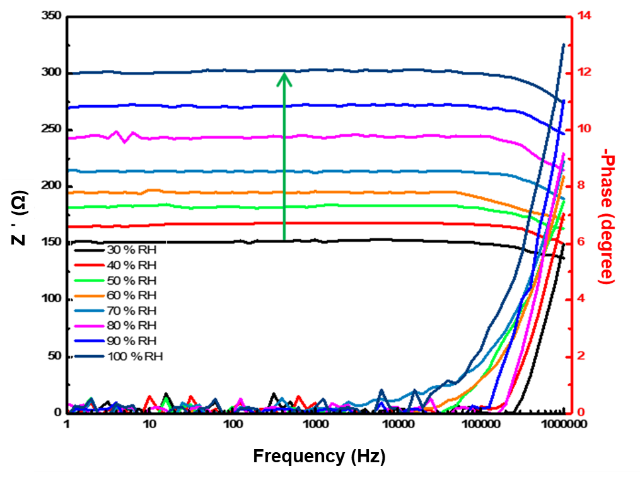


Figure S15. EIS curves of CNT@CPM-3with frequency, when the humidity increases from 30 to 100% RH.


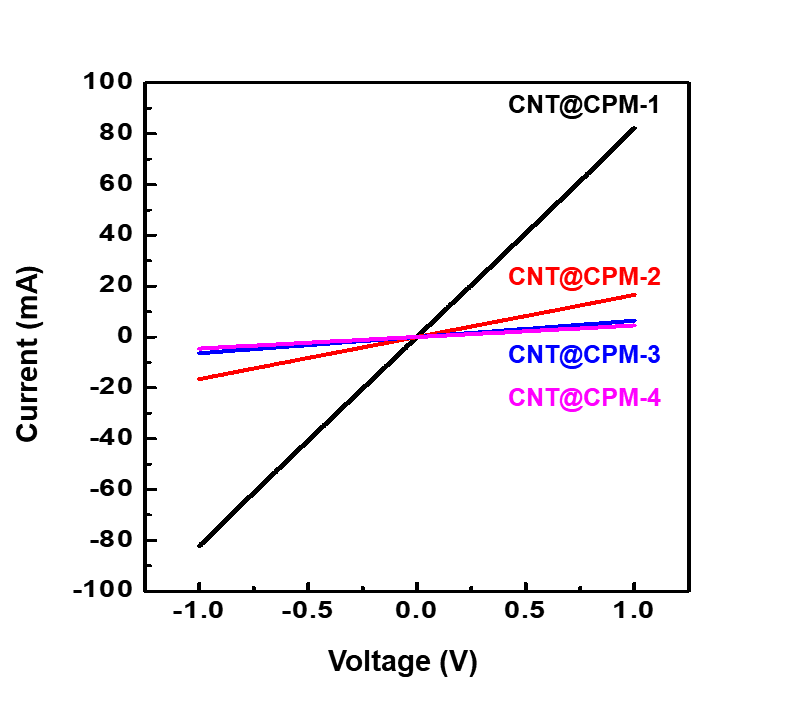


Figure S16. I-V characteristics of the sensors under different PM concentrations.


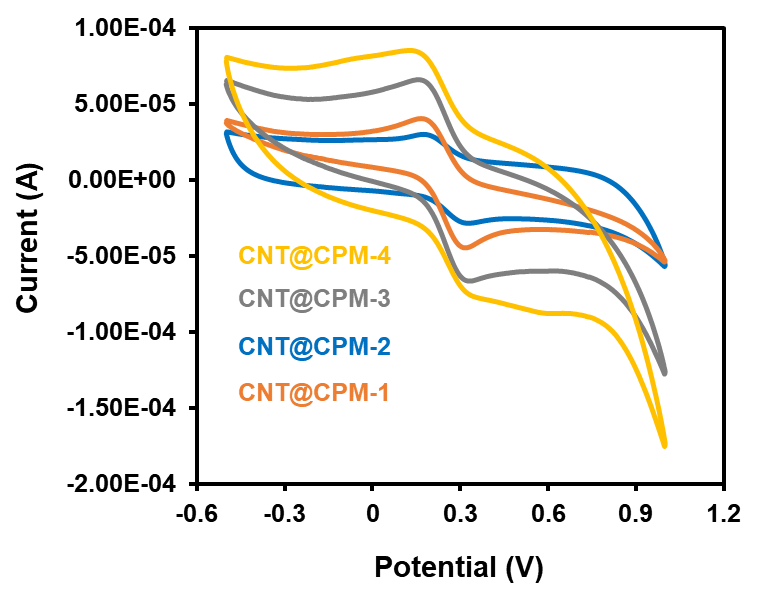


Figure S17. CV characteristics of the sensors under different PM concentrations.


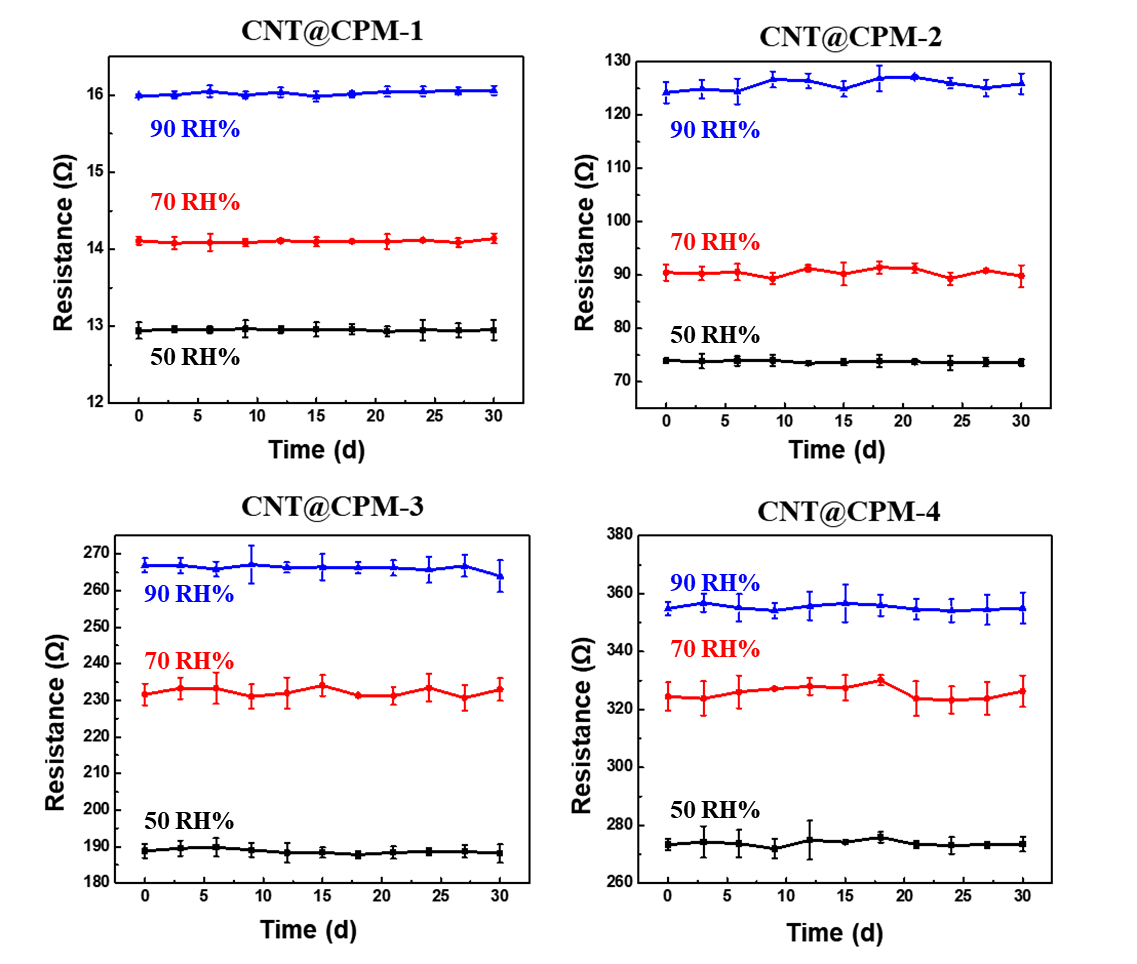


Figure S18. Long-term stability results of the CNT@CPM flexible sensor at 50%, 70%, and 90% RH.


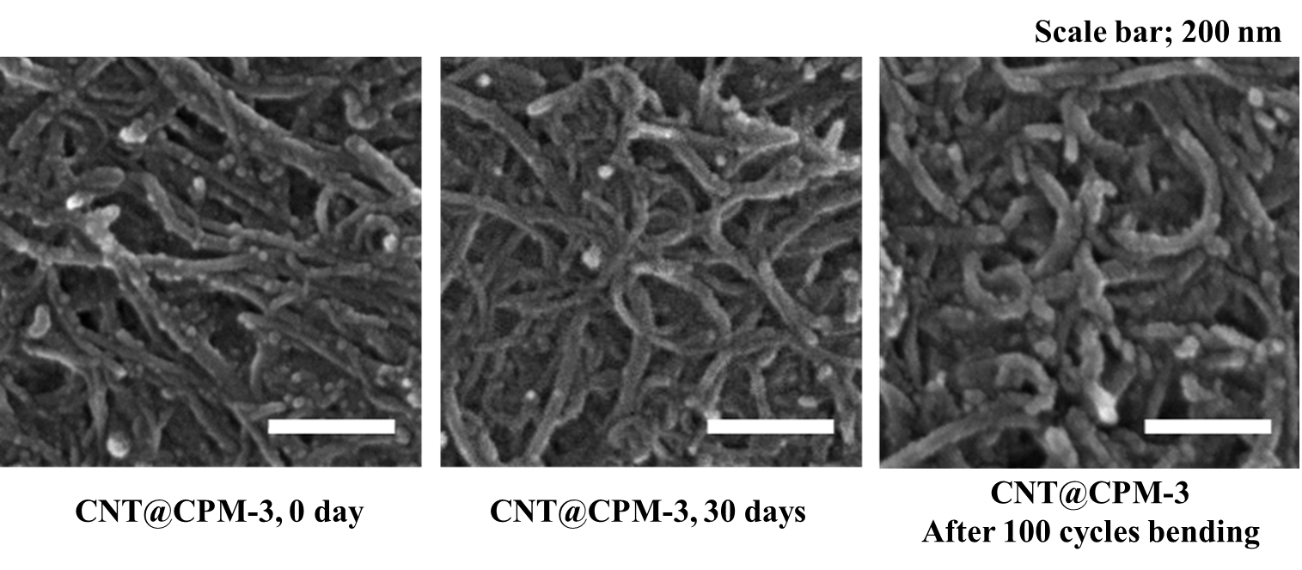


Figrue S19. SEM images of long-term and physical stability of the CNT@CPM.


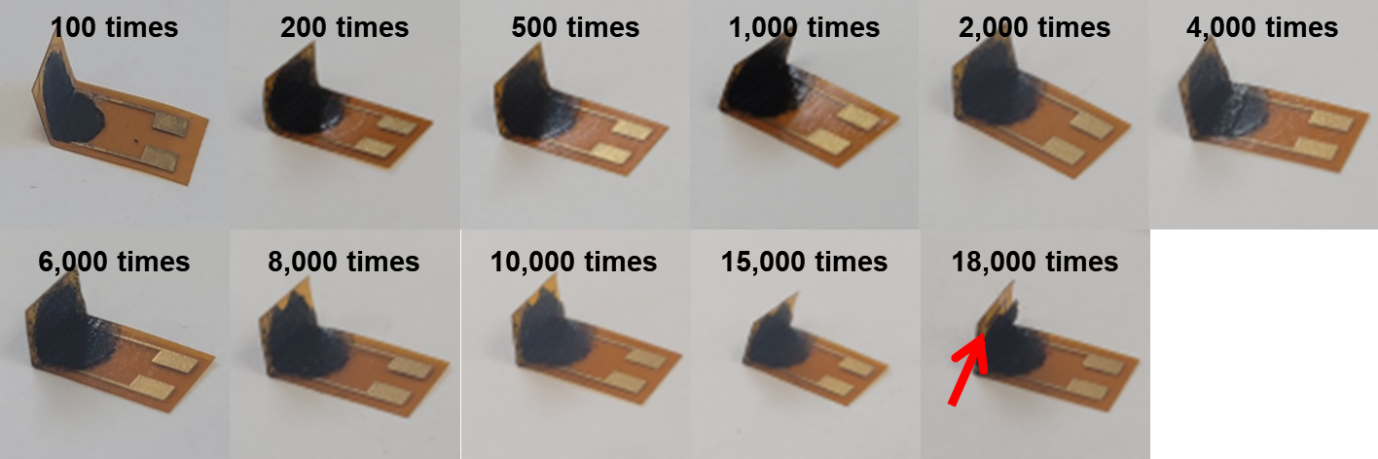


Figure S20. Mechanical durability test photos of sensor under repeated bending cycles.


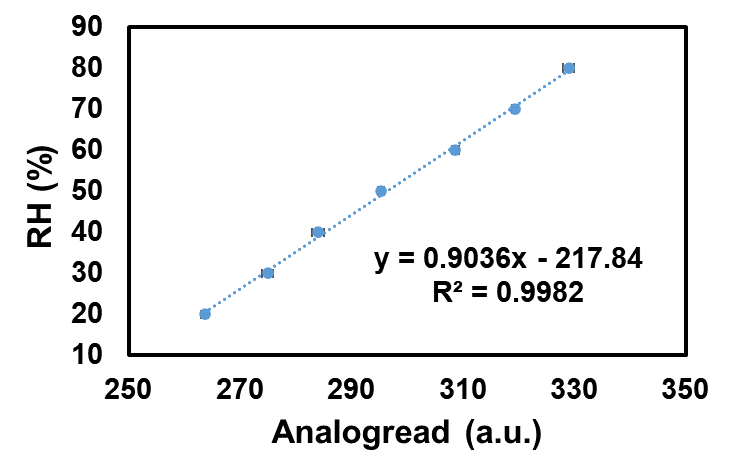


Figure S21. Standard curve of humidity sensing using analog value.


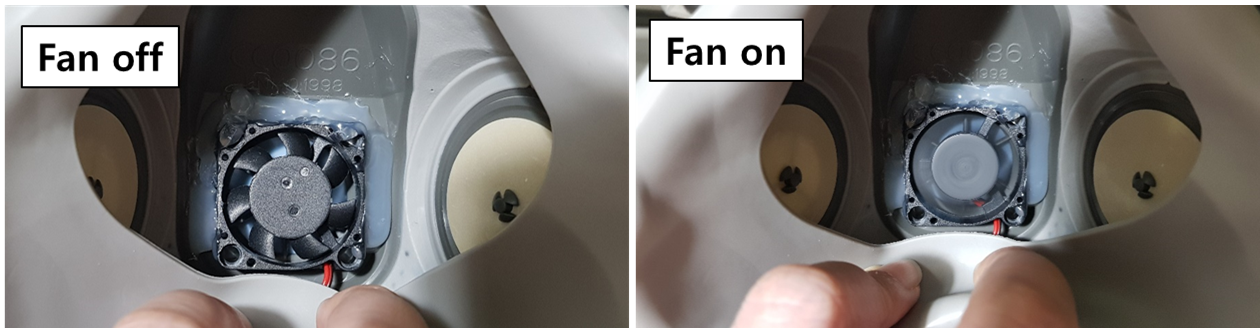


Figure S22. Actual performance images of humidity sensing using face mask.

Video S1. The practical and actual application video of humidity sensor.

Video S2. Actual performance video of humidity sensing and automatic ventilation system.
